# The association of intimate partner violence and contraceptive use: a multi-country analysis of demographic and health surveys

**DOI:** 10.1186/s12939-023-01884-9

**Published:** 2023-04-26

**Authors:** Muluken Dessalegn Muluneh, Lyn Francis, Kingsley Agho, Virginia Stulz

**Affiliations:** 1 Amref Health Africa in Ethiopia, Addis Ababa 17022, Bole Sub, Ethiopia; 2 School of Nursing and Midwifery, Western Sydney University, Parramatta South Campus, Penrith, NSW 2751 Australia; 3 School of Health Sciences, Western Sydney University, Locked Bag1797, Penrith, NSW 2571 Australia; 4 African Vision Research Institute (AVRI), University of KwaZulu-Natal, Durban, 4041 South Africa; 5 School of Nursing and Midwifery, Western Sydney University, Locked Bag 1797, Penrith, NSW 2751 Australia

**Keywords:** Contraceptives, Intimate partner violence, Logistic regression, Eastern SSA

## Abstract

**Background:**

Intimate partner violence (IPV) affects millions of women each year and has been recognized as a leading cause of poor health, disability, and death among women of reproductive age. However, the existing studies about the association between IPV and contraceptive use have been found to be conflicting and relatively less studied, particularly in low and middle income countries, including Eastern Sub Saharan Africa (SSA). This study examines the relationship between IPV and contraceptive use in Eastern SSA countries.

**Methods:**

The Demographic and Health Surveys (DHS) from 2014 to 2017 were a multi-stage cluster sample survey of 30,715 ever married (or cohabitating) women of reproductive age from six countries. The six Eastern SSA datasets were pooled and multivariable logistic regression using a hierarchical approach was performed to examine the association between IPV and contraceptive use after adjusting for women, partners, and household and health facility factors.

**Result:**

Two thirds of women 67% [66.55, 67.88] were not using any modern contraceptive methods and almost half (48%) of the women had experienced at least one form of IPV from their partners. Our analysis showed a strong association with decreased odds of physical violence [adjusted odds ratios (aOR) = 0.72, 95%CI: 0.67, 0 0.78] among women not using any contraceptive methods. Other factors associated with women not using any contraceptive methods were older women (35–49 years), illiterate couples and women from poorest households. Women who had no access to any form of communication [aOR = 1.12, 95%CI: 1.08, 1.36], unemployed partner [aOR = 1.55, 95%CI: 1.23, 1.95] and women who travelled long distances to access health services [aOR = 1.16, 95%CI: 1.06, 1.26] significantly reported increased odds of not using any contraceptive methods.

**Conclusion:**

Our study indicated that physical violence was negatively associated with not using any contraceptive method among married women in Eastern SSA countries. Tailored intervention messages to reduce IPV including physical violence among women not using contraceptive methods in East Africa should target those from low-socioeconomic groups especially, older women with no access to any form of communication, unemployed partners, and illiterate couples.

**Supplementary Information:**

The online version contains supplementary material available at 10.1186/s12939-023-01884-9.

## Background

IPV includes acts of physical aggression, psychological abuse, sexual coercion and controlling behaviours within an intimate relationship [1]. IPV affects millions of women each year and has been recognized as a leading cause of poor health, disability, and death among women of reproductive age [2]. Population-based surveys found 13–61% of women throughout the world reported being physically assaulted by an intimate male partner during their lives from age 15 and 6–59% of women up to 49 years of age had experienced sexual assault by a partner at some point in their lives [3]. Specifically, within SSA countries, 44% of women have been victims of IPV [4].

Reproductive coercion and taking control of women’s reproductive health is one form of IPV (1). Women may be forced to engage in sexual intercourse or practise unprotected sex with their male partners and male partners may sabotage women’s use of contraceptives to increase their female partner’s dependency or to otherwise express their control over their partner’s decision-making [5, 6]. IPV has several effects on women’s physical, mental, and reproductive health [7, 8]. Reproductive health consequences of IPV include unintended pregnancy [9], sexually transmitted infections [10, 11] obstetric complications such as haemorrhage, abortion, and foetal complications [7, 12], and reduced utilization of maternal health services including contraceptive use [13]. Moreover, the effects of IPV are most pronounced in developing countries due to the limited efforts in preventing and supporting women’s recovery who have experienced IPV [9, 14].

A woman’s ability to control her own family planning choices is a critical aspect of her reproductive autonomy and key to increasing contraceptive use and decreasing unintended pregnancy [6]. Therefore, increasing the use of modern contraceptives among women is an important step towards reducing unintended pregnancies and related negative health outcomes [5, 6]. Moreover, contraceptive use is an important part of the United Nations Sustainable Development Goals (SDG) including Goal 3 regarding good health and well-being for all and Goal 5 on promoting equality and empowerment of women and girls [15].

However, the causal mechanisms between the association of women’s autonomy, experiences of IPV and contraceptive use are poorly understood [16]. The review from the existing studies have been found to be conflicting. In some settings, IPV has been found to be associated with a decreased likelihood of modern contraceptive use [17, 18]. However, studies conducted in other settings have found that IPV and contraceptive use are positively associated [19]. The associations between IPV and contraceptive use have been relatively less studied, particularly in low and middle income countries settings including SSA [20]. One of the main reason contradicting each other for studies conducted in SSA focused on small-scale studies [2, 9]. The small-scale studies conducted cannot be generalizable to the wider population. As a result, many SSA countries are yet to include the elimination of IPV on their policy agendas as a serious human rights violation with severe short and long-term implications [21]. Addressing this gap in knowledge can help in identifying the factors affecting contraceptive use among women experiencing IPV.

Understanding how IPV influences women’s ability to adopt contraception is central to designing family planning interventions that enable women who experience IPV to manage their fertility and to inform future interventions. Therefore, the current study assesses the associations of modern contraceptive use with IPV among a population-based sample of married women in Eastern SSA, where IPV is highly prevalent [22, 23].

Various theoretical approaches, views and frameworks have been adopted to explain the potential relation of IPV and other factors including family planning utilisation. The most widely used framework in public health research on IPV is the ecological framework. There are also arguments that there is no single theory that fully explains IPV because the causes are multifaceted, interrelated and contextual with dynamic non-linear pathways [24, 25]. The Ecological model considers the complex interplay between individual, relationship, community, and societal factors that may lead to IPV [26]. The study’s core research questions are: Are the use of contraceptives significantly associated with IPV among women in Eastern SSA countries? What are the other associated factors that affect recent contraceptive use? Improved understanding of these relationships may enhance our identification of risk factors. This, in turn, may inform the development of public health programs to better assist women facing IPV in Eastern SSA countries, as well as other similar settings.

## Methods

### Data source

This study used pooled data of six Eastern SSA countries from DHS which is a nationally representative survey. The countries included were Burundi (2017), Ethiopia (2016), Kenya (2014), Rwanda (2015), Tanzania (2016), and Uganda (2016) [27]. The survey involved the use of a two staged stratified sampling technique [27]. Women in the reproductive age group (15–49 years) were interviewed in each of the six Eastern SSA countries. From the sample households, women from selected households were chosen and completed the DHS domestic violence module. The domestic violence module was randomly administered to only one woman in a household if there was more than one woman living in that household. The unit of analysis for this study were women who were married or were cohabiting in the last five years preceding the survey.

### Outcome variable

Recent contraceptive use was a dependent variable for this study. The DHS surveys on recent contraceptive use were defined as the most recent contraceptive use in the last five years at the time of the survey. It was described as contraceptive use in three categorical options “yes using any modern contraceptive method”; “yes using the traditional contraceptive method” and “never”. As such, for this study, the recent contraceptive use of respondents was categorized as “used modern contraceptive method” and “never/ traditional contraceptive use”. Finally, never or traditional contraceptive use was coded ‘0’ and use of modern contraceptive was coded ‘1’.

### Predictor variable

The primary independent variables in the analysis included lifetime experiences of IPV (physical, sexual or emotional) by their current partner.

Experiences of IPV were the main variables for the analysis. IPV was measured by the experience of at least one or more forms of violence such as physical, sexual or emotional violence. The following were the key questions used to measure IPV in the DHS. Physical violence includes seven dimensions whilst emotional and sexual violence include three questions for each. All the questions were categorical with responses of yes or no. Women were asked whether they had experienced acts of violence within their relationship, perpetrated by their husband / partner. The below questions were categorised as experiencing any violence in their lifetime.

Physical violence experience questions included: (1) push you, shake you, or throw something at you? (2) slap you? (3) twist your arm or pull your hair? (4) punch you with his/her fist or with something that could hurt you? (5) kick you, drag you, or beat you up? (6) try to choke you or burn you on purpose? (7) threaten or attack you with a knife, gun, or any other weapon?

Sexual violence experience questions included: (1) physically force you to have sexual intercourse with him even when you did not want to? (2) physically force you to perform any other sexual acts you did not want to? (3) force you with threats or in any other way to perform sexual acts you did not want to?

Emotional violence experience questions included: (1) say or do something to humiliate you in front of others? (2) threaten to hurt or harm you or someone close to you? (3) insult you or make you feel bad about yourself?

### Explanatory variables

In addition, other factors related to women’s demographic factors (such as age, education, employment status, knowledge of family planning, occupation, religion, alcohol use) are important factors that are associated with contraception utilisation [5, 6, 7]. For instance, as women’s education improved, the likelihood of contraceptive use increased for various reasons [2, 6, 17] including, partner’s factors (e.g. age, education, controlling behaviour, consuming alcohol, physical violence, employment) [12,14], household factors (including wealth, access to information and health facility access) [2, 9, 14], and community factors identified from the literature review [2, 28] were also included in the analysis.

The following variables were defined and recorded as follows:

Decision-making skills of women refers to women participating in decision-making if they make decisions alone or jointly with their partner. This included women who participate in all decision-making about major purchases, health care access and visits to family and relatives. For this analysis, decision-making was categorised as full (participation in all of the decision-making dimensions), considerable participation (at least in one) and no decision-making (no participation in any decision-making).

Wealth index is a composite measure of a household’s cumulative living standard. The wealth index is calculated using easy-to-collect data on a household’s ownership of selected assets, such as televisions and bicycles; materials used for housing construction; and types of water access and sanitation facilities. DHS separates all interviewed households into five wealth quintiles (very poor, poor, medium, rich and richest). In this analysis we merged poorest and poor which was categorised as” poor”, and rich and richest was categorised in one group” rich”.

Justification or acceptance of women towards physical violence was dichotomized (justified/not justified). Women’s acceptance towards physical violence was measured by computing the following variables (burning food, arguing with the husband, going out without telling the husband, neglecting the children, and refusing to have sexual intercourse with the husband). If women responded “yes” to at least one of the above five variables, they were considered to accept physical violence (See Supplementary Table A).

### Analysis

The study used the Svyset command to account for a complex survey design and to provide unbiased estimates for odds ratios (OR) and their confidence intervals (CI’s). STATA version 16.0 was used for analysis. A chi-square test was used to assess the association of IPV and other covariates with current contraceptive use. Multi-collinearity of the independent variable was checked before running multivariable models. Hierarchical logistic regression analysis was used to estimate the effects of various forms of IPV and other factors on contraceptive use. The analysis used ecological tool as an explanatory tool for analysis. To avoid an excessive number of variables and unstable estimates in the subsequent model, only variables that reached a p-value less than 0.05 were kept in the subsequent analyses except the predictor variable (22). The final models for analysis underwent the following processes to fit the final model. Model 1: Estimates of the effects of IPV (Physical, sexual and emotional violence) on contraceptive use; Model 2: The effects of selected demographic factors of women on contraceptive use; Model 3: The effects of selected household level demographic associated factors on contraceptive use; Model 4: The effects of selected partner level factors on contraceptive use. Model 5: The effects of selected community and health facilities factors on contraceptive use. Only covariates that showed significance in the model (2 to 5) (p < 0.05) and all forms of IPV were included in the final model. The final model involved the combined effects of all from IPV and all other associated factors associated with contraceptive use (See Table 1).

**Table 1:** Potential covariates used for hierarchical survey logistic regression model

Model 1- IPV factors	Model 2: Model 1 + Woman demographic factors	Model 3: Model 1 + Household factors	Model 4: Model 1 + Partners factors	Model 5- Model 1 + community and barriers to health care factors	Model 6 = Model 1toModel 5
Physical violence, sexual violence, emotional violence and sought help after IPV	Woman’s age, first sexual intercourse, education, marital status, living number of children, women reported being justified for violence, women drinking alcohol	Head of household, wealth index, women’s access to communication	Age of partner, number of children, partner’s employment status, partner’s education, women decision making index, history of woman’s father being physically violent, partner exerted controlling behaviours	Seek permission to visit health services , getting money to pay health services, hesitancy of attending health care alone travelling long distance to get health service, country and place of residence	All factors from Model 2 to 5 with significant at p < 0.05)

### Ethical clearance

All DHS surveys are approved by the Inner City Fund (ICF) international and an institutional review board in specific countries to ensure that they comply with the United States Department of Health and Human Services’ regulations to protect human subjects. Additionally, the Western Sydney University Ethics Committee has approved the research protocol. In all phases, the data included in this analysis contain no identifying information and are publicly available.

## Results

### Descriptive results of current family planning use and IPV in Eastern SSA countries

This study covered the six Eastern SSA countries’ DHS data with a weighted sample of 30,715 married or cohabiting women conducted between 2014 and 2017. Table 2 shows the different study variables using both weighted and unweighted frequencies and proportions.

**Fig. 1:**
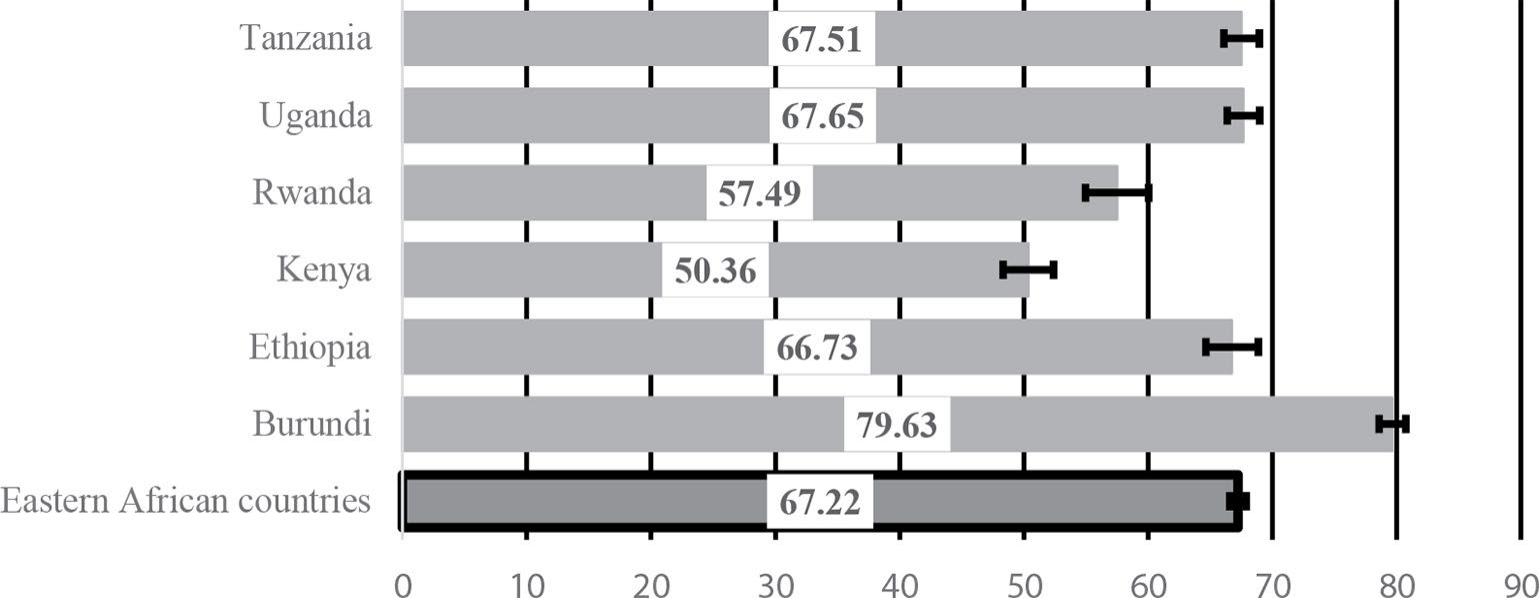
Prevalence and 95% confidence intervals (CI) of non-use of contraceptives

Figure 1 revealed that only a third of women were current users of family planning in Eastern SSA, and that more than two-thirds of women (67.22% [66.55, 67.88]) were not using family planning at the time of the surveys. The contraceptive utilisation rate was lowest in Burundi at 20% [19.32, 21.47] and relatively higher in Kenya (49.6% [47.6, 51.67], that has better contraceptive utilisation in comparison to other Eastern SSA countries

**Table 2:** Characteristics of the study variables in Eastern SSA countries from 2014–2017

Variables	Unweighted (n = 33,640) %)	Weighted (n = 30,715) %) *
IPV factors		
**Any type IPV experience**		
Yes	15,637(46.5)	14,737(48)
**Physical violence**		
Yes	11,911(35.4)	11,218(36.5)
**Sexual violence (n = 33,637)**		
Yes	5,643(16.8)	5,384(18)
**Emotional violence**		
Yes	10,408 (30.9	9,879(32.2)
Community level factors		
**Country**		
Burundi	7,366 (21.9)	6,558(21.4)
Ethiopia	4,720(14.0)	4,469(14.6)
Kenya	4,515(13.4)	4,018(13.1)
Rwanda	1,906(5.7)	1,689(5.5)
Uganda	7,536(22.4)	6,879(22.4)
Tanzania	7,597(22.6)	7,101(23.1)
**Place of residence**		
Urban	8,056(23.9)	7313(23.8)
Rural	25,584(76.1)	23,402(76.2)
Household factors		
**Head of household**		
Male	24,901(74)	22,826(74.3)
Female	8,739 (26%)	7,889 (25.7)
**Household Wealth Index**		
Rich	12,887(38.3)	11,890(40.7)
Middle	6,228(18.5)	5,974(19.5)
Poor	14,525(43.2)	12,851(39.8)
**Use of any of communications**		
Yes	22,393(66.6)	20,815(67.8)
No	11,247(33.4)	9,900(32.2)
**Decision-making power (n = 33,612)**		
Full decision	1,534(4.6)	1,384 (4.5)
Considerable	4,349 (12.9)	3,866(12.6)
No power	27,729(82.5)	25,465(82,8)
**Woman reported acceptance of violence (n = 33,627)**		
Not Justified	14,916(44.4)	13,200 (43.7)
Justified	18,711(55.6)	17,515(56.3)
Women’s demographic factors		
**Woman’s age**		
15–24	7,669(22.8)	6,803(22.2)
25–34	14,244(42.3)	12,207(39.7)
35–49	11,727(34.9)	11,704(38.1)
**Woman’s age at first marriage**		
18–49 years	20,052(59)	18,137(59)
15–18 years	13,588 (41)	12,578(41)
**Age at first sexual experience (years)**		
< 15	4,415(13.1)	4,151(13.5)
15–18	17,602 (52.3)	16,622(54.1)
> 18	11,623(34.6)	9,942 (32.4)
**Woman’s education**		
Secondary and above	6,220 (18.5)	5,738 (18.7)
Primary	17,532(52.1)	16,143 (52.6)
No education	9,888(29.4)	8,833(28.7)
**Woman’s employment status (n = 33,637)**		
Working	25,010 (74.4)	22,978 (74.8)
Not working	8,627(25.6)	7,735 (25.2)
**Marital status (n = 32,553)**		
Married	21,371(85.6)	19,953 (63.7)
Widowed/divorced	8,955(27.5)	8,242 (28.8)
Separated	2,227(6.8)	2,520 (8.5)
**Current marital status**		
currently	28,893 (85.9)	25,724 (83.8)
formerly	4,747 (14.1)	4,991 (16.2)
**Number of times married (n = 33,630)**		
One	28,577 (85)	26,065 (84.9)
more than one	5,053(15)	4,650 (15.1)
**Woman’s religion (n = 25,518)**		
Muslim	3,845(15.1)	2,870 (12.4)
Catholic	9,022(35.4)	7,953(34.4)
Orthodox	1,810(7.1)	19,05(8.2)
Protestant	6,997 (27.4)	6,875(29.7)
Angelica	2326(9.1)	2,149(9.3)
Seventh day	574(2.3)	477(2.1)
Pentecostal	944(3.7)	890(3.9)
**Number of children ever born**		
0–2	12,687(37.7)	11,060(37.6)
3–5	13,198(39.3)	12,051(37.7)
6 or more	7,755(23.1)	7,604(24.7)
**Number of living children**		
0–2	13,936 (41.4)	13,010 (41.2)
3–5	13,995(41.6)	12,025(40.3)
6 or more	5,709(17)	5,680 (18.5)
**Knowledge of any type of family planning (FP) method**		
Modern methods	33,204(98.7)	30,507(99.3)
No method	436(1.3)	208(0.7)
**Ever used FP method**		
Yes used	20,250 (60.2)	19,427 (63.2)
Never used	13,390(39.8)	11,288 (36.8)
Partner’s Factors		
**Partner’s age**		
15–24	1,999(5.9)	1,758(5.7)
25–35	10,552(31.4)	8,934(29.1)
36–50	13,171(39.2)	11,871(38.7)
> 50	7,918(23.5)	8,152(26.5)
**Partner’s employment status (n = 23,398)**		
Working	22,339(95.5)	22,500 (95.8)
Not working	1,059(4.5)	890(4.2)
**Partner’s education (n = 29,541)**		
Secondary and above	7,310(24.8)	6616(25)
Primary	15,547(52.6)	17,224(53.4)
No education	6,684(22.6)	5701(21.6)
**Partner consumes alcohol**		
No	18,816 (55.9)	17,140 (55)
Yes	14,823(44.1)	13,575(45)
**Father physically violent towards her mother (n = 33,639)**		
No	19,888(59.1)	17,952 (58.5)
Yes	13,746(40.9)	12,752.41.5)
**Controlling behaviour of partner (n = 33,626)**		
No	14,016(41.7)	11,855(39.7)
Yes	19,610(58.3)	18,860(60.3)
Barriers to woman’s health care		
**Seek permission to visit health services (n = 33,639)**		
Not a big problem	30,134 (89.6)	27,237(88.7)
Big problem	3,505 (10.4)	3478(11.3)
**Accessing money to pay health services (n = 33,638)**		
Not a big problem	15,664(46.6)	15,000 (47.3)
Big problem	17,974(53.4)	15,715(52.7)
**Travelling long distances to access health services (n = 33,637)**		
Not a big problem	20,645(61.4)	17,722 (61.3)
Big problem	12,992(38.6)	12,993(38.7)
**Hesitancy of attending health care alone (n = 33,636)**		
Not a big problem	25,846(76.8)	23,381(76.1)
Big problem	7,790(23.2)	7334 (23.9)

*Weighted for the sampling probability, weighted total was 30,715 otherwise stated within brackets

**Fig. 2:**
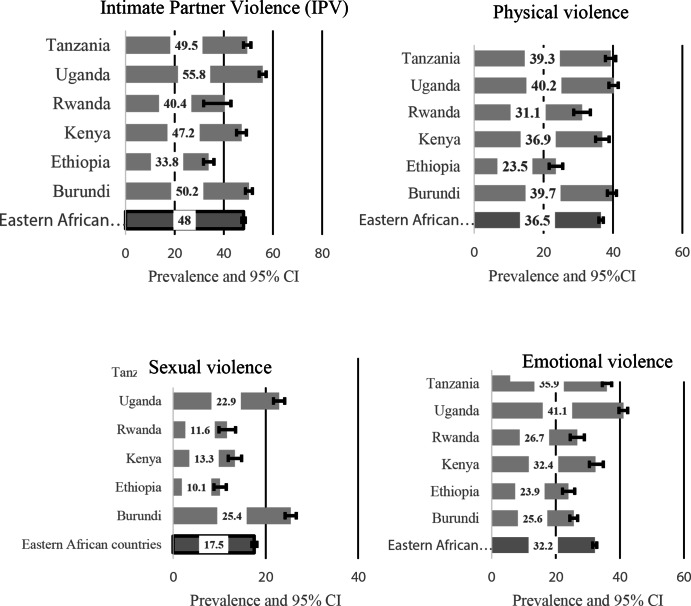
Prevalence and 95% confidence intervals of IPV (Physical, sexual, or emotional violence)

In Eastern SSA, almost half (48%) of the women have experienced at least one form of IPV from their partners. The prevalence varies by country, where Uganda (56%) and Burundi (50%) and Tanzania (49.5%) were the top three countries with the highest proportion of women who reported IPV (See Fig. 2).

### Factors associated with contraceptive use including IPV and current contraceptive use in Eastern SSA countries

This study showed there was an association between current contraceptive use and IPV. The Pearson chi-square associations showed women who had experienced any form of IPV were more likely to use family planning at the time of the survey in comparison to women who did not experience any form of IPV (See Table 3). In the bivariate analysis, women who experienced physical violence were more likely to use contraceptives in comparison to women who had experiences of sexual or emotional violence.

Overall, the results have revealed that family planning use is associated with women’s age, women’s education status, marital status, number of times being married, religion and the number of living children. For example, contraceptive use among illiterate women was more than 10% lower than those women who had secondary or higher education levels in Eastern SSA countries. Similarly, contraceptive utilisation was associated with a female-headed household, women being categorised as a rich socioeconomic class, women who did not accept physical violence, and those women who have access to social media or other communication outlets. For instance, women in the rich wealth quartile had relatively higher contraceptive utilisation (37.84% [36.68, 39.01] in comparison to women in the poor wealth quartile 26.88% [25.97, 27.8] (See Table 3).

**Table 3:** Prevalence of never or traditional use of contraceptive by IPV, community, household, women demographics, partner and barriers to woman’s health care factors in Eastern SSA countries

Variables	Proportion of contraceptive use (Never or traditional used)	UOR [95% CI]	P value
IPV factors			
**Any form of IPV**			
No	68.34[67.42,69.25]	1.00	
Yes	66[65.03,66.96]	0.89[0.85, 0.96]	< 0.001
**Physical violence**			
No	68.5[67.7,69.35]	1.00	
Yes	64.94[63.81,66.05]	0.85[0.79, 0.91]	< 0.001
**Sexual violence**			
No	66.92[66.18,67.65]	1.00	
Yes	68.62[67.06,70.14]	1.1[0.99,1.17]	0.053
**Emotional violence**			
No	68.12[67.32,68.91]	1.00	
Yes	65.32[64.1,66.51]	0.88[0,83,0.94]	< 0.001
**Sought help after IPV**			
No	67.53[66.31,68.73]	1.00	0.003
Yes	64.64[63.13,66.11]	0.87[0.81, 0.95]	
Community level factors			
**Place of residence**			
Urban	59.78[58.18,61.36]	1.00	
Rural	69.54[68.82,70.25]	154[1.43, 1.65]	<0.001
**Country**			
Burundi	79.63[78.53,80.68]	1	
Ethiopia	66.73[64.59,68.79]	0.51[0.46 0.57]	< 0.001
Kenya	50.36[48.33,52.4]	0.26[0.23,0.29]	
Rwanda	57.49[54.93,60]	0.34[0.31,0.39]	
Uganda	67.65[66.33,68.94]	0.53[0.48,0.59]	
Tanzania	67.51[66.07,68.91]	0.53[0.48,0.58]	
Household Factors			
**Head of household**			
Male	65.29[64.51,66.06]	1.00	
Female	72.81[71.5,74.08]	1.42[132,1.53] ]	< 0.001
**Household wealth index**			
Rich	62.16[60.99,63.32]	1.00	
Middle	65.67[64.17,67.15]	1.16[1.07,1.26]	< 0.001
Poor	73.12[72.2,74.03]	1.65[1.54, 1.77]	< 0.001
**Use of any communications means**			
Yes	63.5[62.66,64.33]	1.00	
No	67.22[73.95,76.1]	1.72[1.61, 1.85]	< 0.001
**Decision-making power index**			
Full decision making power	63.19[59.99,66.28]	1.00	
Considerable decision making power	59.75[57.8,61.68]	0.86[0.74, 1.01]	<0.001
No decision making power	68.57[67.84,69.29]	1.27[0.1.10, 1.46]	
**Woman not accepting physical violence**			
Not Justified	64.76[63.72,65.79]	1.00	
Justified	69.12[68.25,69.99]	1.22[1.15, 1.29] ]	< 0.001
Woman’s demographic factors			
**Woman’s age**			
15–24	69.03[67.65,70.38]	1.00	
25–34	62.83[61.78,63.87]	0.76[0.70, 0.82]	< 0.001
35–49	70.74[69.62,71.84]		
**Woman’s age at first marriage**			
18 and above	67.35[66.48,68.2]	1.00	
less 18	67.03[65.97,68.08]	0.98[0.93, 1.04]	0.652
**Age at first sexual experience (years)**			
< 15	66.59[64.71,68.43]	1.00	
15–18	66.1[65.17,67.01]	0.98[0.89, 1.07]	
> 18	67.22[68.22,70.46]	1.14[1.03, 1.25]	< 0.001
**Woman’s education**			
Secondary and above	59.28[57.52,61.03]	1.00	
Primary	64.64[63.74,65.54]	1.26[1.16, 136]	< 0.001
No education	67.22[75.92,78.2]	2.31[2.09, 2.55]	< 0.001
**Woman’s employment status**			
Working	66.72[65.96,67.48]	1.00	
Not working	68.69[67.28,70.05]	1.09[1.02, 1.18]	0.016
**Marital status**			
Married	64.92[64.06,65.78]	1.00	
Widowed/divorced	69.67[68.46,70.84]	1.24[1.16, 1.33]	< 0.001
Separated	73.75[71.27,76.1]	1.52[1.33, 1.73]	< 0.001
**Number of children ever born**			
currently	65.33[64.61,66.05]	1.00	
Formerly	76.93[75.26,78.53]	1.77[1.60, 1.95]	< 0.001
**Number of times married**			
One	66.66[65.93,67.38]	1.00	
more than one	70.35[68.68,71.97]	1.19[1.09, 1.29]	< 0.001
**Religion denomination**			
Muslim	74.[72.56,76.97]	1.00	
Catholic	70.21[69.04,71.36]	0.79[0.69, 0.90]	< 0.001
Orthodox	60.74[57.48,63.91]	0.52[0.44, 0.62]	< 0.001
Protestant	62.24[60.74,63.72]	0.55[0.48, 0.63]	< 0.001
Angelica	64.42[62,66.77]	0.61[0.52, 0.71]	< 0.001
Seventh day	66.97[62.46,71.19]	0.68[0.54, 0.85]	< 0.001
Pentecostal	67.8[64.08,71.31]	0.71[0.58, 0.87]	< 0.001
**Number of children ever born**			
two or less	68.45[67.35,69.52]	1.00	
three to five	63.12[62.03,64.19]	0.79[0.74,0.85]	< 0.001
greater than six	71.59[70.24,72.9]	1.16[1.06, 1.26]	< 0.001
**Number of living children**			
two or less	68.72[67.68,69.74]	1.00	
three to five	63.72[62.67,64.75]	0.79[0.75,0.85]	< 0.001
greater than six	71.51[69.91,73.06]	1.14[1.04, 1.25]	< 0.001
Partners’ factors			
**Partner’s age**			
15–24	74.02[71.56,76.34]	1.00	
25–35	62.92[61.72,64.11]	0.59[0.52,0.68]	< 0.001
36–50	63.7[62.6,64.79]	0.62[0.54,0.70]	< 0.001
> 50	75.58[74.26,76.86]	1.09[0.94. 1.25]	
**Partner’s employment status**			
working	68.62[67.81,69.41]	1.00	
not working	78.62[74.74,81.73]	1.66[1.25, 2.05]	< 0.001
**Partner’s education**			
Secondary and above	59.33[57.71,60.92]	1.00	
Primary	64.28[63.33,65.22]	1.23[1.14, 1.33]	< 0.001
No education	75.61[74.15,77.02]	2.13[1.92, 2.35]	< 0.001
**Partner consuming alcohol**			
No	66.76[65.82,67.69]	1.00	
Yes	67.79[66.84,68.72]	1.05[0.98, 1.11]	0.131
**Father physically violent towards her mother**			
No	67.86[66.98,68.73]	1.00	
Yes	66.33[65.28,67.35]	0.93[0.88, 0.99]	< 0.05
**Partner’s controlling behaviour**			
No	70.74[69.75,71.71]	1.00	
Yes	64.91[64.01,65.8]	0.76[0.72, 0.81]	< 0.001
Barriers to woman’s health care			
**Seek permission to visit health services**			
Not a big problem	66.8[66.1,67.49]	1.00	
Big problem	70.48[68.29,72.58]	1.34[1.26, 1.43]	< 0.001
**Accessing money to pay health services**			
Not a big problem	63.81[62.78,64.82]	1.00	
Big problem	70.28[69.41,71.15]	1.27[0.19, 1.35]	< 0.001
**Travelling long distances to access health services**			
Not a big problem	65.19[64.33,66.05]	1.00	
Big problem	70.42[69.37,71.45]	1.27[1.19, 1.35]	< 0.001
**Hesitancy of attending health care alone**			
Not a big problem	66.31[65.54,67.06]	1.00	
Big problem	70.11[68.71,71.47]	1.19[1.11,1.28]	<0.001

Χ^2^-test was applied to test statistical significance for the prevalence estimates. UOR = unadjusted odds ratios. Odds ratios (ORs) statistically greater that 1.00 indicate risk factors and those statistically smaller than 1.00 are protective factors. 95% confidence intervals (CI) around ORs that lies between 1.00 indicate not statistically significant.

Younger age, women’s higher education status, employment, partners’ controlling behaviour and partners consuming less alcohol were associated with women’s increased recent contraceptive utilisation in Eastern SSA countries. Similarly, current contraceptive non-use was associated with barriers to health care due to lack of permission from partner, travelling long distances, having no money and lack of an accompanying support person ( P < 0.001) (See Table 3). In addition, women who resided in rural residences had a 10% less chance of contraceptive use in comparison to women residing in an urban residence in Eastern SSA countries.

### Multiple logistic regression results

#### Association between IPV and non-use of contraceptive

The analysis of hierarchical logistic regressions showed women who were exposed to physical violence were more likely to use contraceptives 0.72 [0.67, 0 0.78]. Alternatively, there were no significant differences in contraceptives use among women who were exposed to sexual and emotional violence in comparison to those who were not exposed.

#### Factors associated with non-use of contraceptive

In addition, not using contraceptives was associated with: women who were older 0.84 [0.74, 0 0.95], women with older partners 0.77 [0.65, 0.91], women with little or no education 1.49 [1.31, 1.69], women with an illiterate partner 1.19 [1.04, 1.34], women who had poor wealth quintiles 1.34 [1.23, 1.46], women with no communication access 1.19 [1.09, 1.29], women who had barriers gaining permission 1.17 [1.03, 1.31] and women with no money to access health care 1.11 [1.03, 1.19] (p < 0.001). For instance, women with higher socioeconomic status were 1.34 times more likely to use contraceptives in comparison to women with lower socioeconomic status. Similarly, women who had experienced physical violence had a 28% higher chance of using contraceptives in comparison with those who did not experience physical violence (See Table 4).

**Table 4:** Multiple logistic regression analysis of adjusted odds ratio (AOR) of IPV, individual, household and partner variables associated factors of contraceptive non-use amongst married women of 15–49 years of age in Eastern SSA countries

Variables	Model 1	Model 2	Model 3	Model 4	Model 5	Final Model
	AOR [95% CI]	AOR [95% CI]	AOR [95% CI]	AOR [95% CI]	AOR [95% CI]	AOR [95% CI]
IPV factors						
**Physical violence**						
Yes (No, OR = 1.00)	0.83[[0.75,0.92]	0.79[0.73, 0.85]	0.79[0.73, 0.855]	0.88[0.8, 0.96]	0.78[0.73,0,85]	0.78[0.71, 0 0.85]
**Sexual violence**						
Yes (No, OR = 1.00)	1.20[1.09,1.31]	1.15[1.06, 1.27]	1.18[1.08, 1,29]	1.17[1.05,1.29]	1.05[0.96,1.16]	1.06[0.96, 1.18]
**Emotional violence**						
Yes (No, OR = 1.00)	0.92[[0.84,1.00]	0.91[0.84, 0.99]	0.92[0.84, 0.99]	0.92[0.83, 1.01]	0.97[0.89,1.95]	0.94[0.85,1.04]
Woman demographic factors						
**Sought help after IPV**						
Yes (No, OR = 1.00)	0.92[0.84,1.00}					
**Woman’s age (years)**						
25–34 (15–24, OR = 1.00)		0.77[0.70, 0.85]				0.87[0.77, 0.98]
35–49		1.01[0.91,1.13]				0.94[0.79, 1.10]
**Woman’s age at first sexual experience**						
15 to 18 (< 15, OR = 1.00)		1.08[0.98, 1.18]				0.95[0.85,1.06]
19 to 49		1.36[1.23,1.52]				1.24[1.08,1.43)
**Woman’s education**						
Primary (secondary or more, OR = 1.00)		1.33[1.22, 1.45]				1.20[1.04,1.33]
No education		2.52[2.27, 2.78]				1.59[1.37,186]
**Woman’s employment status**						
Not working (Working, OR = 1.00)		1.04[0.96, 1.12]				
**Marital status**						
Widowed/divorced (Married, OR = 1.00)		1.30[1.21, 1.39]				0.93[0.85,1.106]
Separated		1.66[1.45, 1.90]				1
**Number of living children**						
3 to 5 children (0–2 children, OR = 1.00)		0.75[0.69, 0.82]				0.71[0.63,0.78]
6–12		0.92[0.82, 1.03]				0.83[0.72,0.96]
**Woman accepting physical violence**						
Justified (Not Justified, OR = 1.00)			1.12[1.05, 119]			1.08[0.99,1.17]
Household related factors						
**Head of household**						
Male (Female, OR = 1.00)				1.40[1.30,1.51]		1.21[1.08,1.36]
**Household wealth status**						
Middle (Rich, OR = 1.00)				1.11[1.01,1.19]		1.01[0.89, 1.12]
Poor				1.4[1.33,1.54]		1.31[1.18, 1.47]
**Any communication means**						
No (Yes, OR = 1.00)				1.53[1.43,1.64]		1.12[1.02, 1.22]
Partners’ factors						
**Partner’s age (years)**						
25–35 years (15–24 years, OR = 1.00)			0.65[0.56,0.75]			0.78[0.66, 0.93]
36–50 years			0.63[0.54,0.73]			0.83[0.68, 1.00]
above 50			0.86[0.71,1.04]			1.09[0.87, 1.38]
**Partner’s employment status**						
Not working (Working, OR = 1.00)			1.56[1.25,1.91]			1.55[1.23, 1.95]
**Partner’s education status**						
Primary (Secondary and above, OR = 1.00)			1.18[1.08,1.30]			0.95[0.86, 1.04]
No education			1.64[1.45,1.84]			1.19[1.04, 1.34]
**Decision-making power**						
Full			1.00			
Considerable power			0.88[0.73, 1.08]			
No power			0.97[0.82, 1.08]			
**Father physically violent towards her mother**						
Yes (NO, OR = 1.00)			0.96[0.69,0.81]			1.08[0.99, 1.17]
**Partner’s controlling behaviour**						
Yes (NO, OR = 1.00)			0.75[[0.69,0.81]			0.88[0.80,0.95]
Barrier to woman’s health care						
**Seek permission to visit health services**						
Big problem (Not a big problem, OR = 1.00)					1.08[0.96, 1.21]	
**Accessing money to pay health services**						
Big problem (Not a big problem, OR = 1.00)					1.11[1.04,1.19]	1.01[0.93,1.09]
**Travelling long distances to access health services**						
Big problem (Not a big problem, OR = 1.00)					1.10[109, 1.18]	1.16[1.06,1.26] ]
**Hesitancy of attending health care alone**						
Big problem (Not a big problem, OR = 1.00)					1.02[0.93, 1.11]	
Community factors						
**Country**						
Ethiopia (Burundi, OR = 1.00)					0.50[0.44,0.56]	0.47[0.41, 0.54]
Kenya					0.28[0.26, 0.32]	0.18[0.13, 0.23]
Rwanda					0.36[0.31,0.41]	0.23[0.19,0.31]
Uganda					0.55[0.51,0.61]	0.78[0.69, 0.88]
Tanzania					0.56[0.51,0.61]	0.79[0.70, 0.89]
**Place of residence**						
Rural (Urban, OR = 1.00)					1.27[1.18,1.37]	1.07[0.95,1.19]

95% confidence intervals (CI) around AORs that lies between 1.00 indicate not statistically significant

## Discussion

This study has explored current contraceptive use and IPV among a large representative sample of married or cohabiting women in Eastern SSA countries using the ecological model as an explanatory tool. IPV affects millions of women each year and has been recognized as a leading cause for poor health, disability, and death among women of reproductive age [2].

In this study, there were a variability in IPV prevalence in Easter SSA countries and this will affect the utilization of decision making on the use of contraceptive in those countries. This was clearly explained by a previous studies using ecological model approach study. For instance, a systematic review and meta-analysis study conducted in SSA countries shows a plethora of information on the overall associated factors that augment the occurrences of IPV in SSA countries [29]. This systematic review has identified that women’s experiences of IPV in SSA are associated with many factors that are related to individual, interpersonal, community and societal levels. Pooled meta-analyses revealed that low educational attainment, higher alcohol consumption, substance use, history of child and family abuse, limited decision-making skills, experiencing depression, males having multiple sexual partners, and younger age were found to be individual- and family-associated factors that increase the experiences of IPV [29]. Community tolerant attitudes to violence, women’s unemployment, being Muslim, lower socioeconomic class, food and social insecurity were found to be community- and societal-associated factors of IPV [29]. Alcohol consumption, low educational attainment, experiencing depression, being younger, a history of child and family abuse, tolerant attitudes to violence, and low socioeconomic status were poignant factors associated with IPV amongst women in SSA countries [29]. Those factors difference in Easter SSA countries affects the occurrence IPV and it will affect directly and indirectly affect the utilization of contraceptive.

Investing in family planning is one of the smart investments for development programs as population dynamics have a fundamental influence on the SDGs [15, 30]. This study has shown that women who currently used contraceptives were significantly associated with women’s experiences of physical violence during their lifetime. This is consistent with previous research in many developing and developed countries including New Zealand [19, 31–34] that identified violence increased contraceptive use.

In analysing data from the ten DHS national surveys from all world regions, Hindin, Kishor, and Ansara [33] found that in seven out of ten countries, ever having used contraception was positively associated with IPV. In the detail, analysis the short acting family planning were more associated with IPV as compared to the long acting family planning including injectables, and pills and emergency contraceptives. This might be related to the following reasons: (i) Women who had an experience of violence might be more likely to use contraceptives because of fears of living with a violent partner (27). Most women might expect to leave their marriage to reduce the risk of pregnancy and caring for an extra child with increased risk of physical violence from their partner (27–29). One possible explanation for higher contraceptive use that has been suggested is that women in abusive relationships may attempt to prevent pregnancy because they do not want to bring a child into a violent family setting [32, 33]. However, their use of contraceptives may be hidden from their partner; (ii) and/or women may have a heightened awareness of their risk of an unintended pregnancy or sexually transmitted infections. In contrast, many other studies showed an opposite relation between contraceptive use and IPV with women who experienced IPV being less likely to use contraceptives [2, 35–37].

Similarly, contraceptive utilisation decreased with older women. This may be due to the fact that younger women have sufficient information or awareness about contraceptive use and the benefits of family planning in comparison with older women who are less educated. Additionally, older women may have enough children and not want any more children in comparison to younger women [38–40].

Younger women who engaged in sexual intercourse before the age of 18 years were less likely to use contraceptives in comparison to those who commenced having sexual intercourse after the age of 18. This may be related to women who engage in sexual intercourse at younger ages being more likely to engage in risky behaviours. This finding is consistent with other studies that showed early intercourse has been associated with engaging in risky behaviours that included not using condoms or contraceptives that subsequently resulted in unintended pregnancies [41, 42].

In our study, women who were more educated were more likely to use contraceptives in comparison to those who were illiterate. This finding is consistent with a study conducted by WHO and others [2, 36, 37]. Most interestingly, women who had a higher education level had a 51% higher chance of using contraceptives in comparison to illiterate women in Eastern SSA countries. Women who were illiterate were 1.49 times more likely not to use contraceptives in comparison with women who had a higher education level. Women who are more educated are more likely to have a better understanding of reproductive health and contraceptive use which may be related to knowledge, awareness and attitudes, which drives the demand for contraceptive methods. This demand may evoke pressure on reproductive health service providers including governments to provide family planning commodities [43, 44].

This study showed the disparity of contraceptive use among various wealth quintiles. The inequality is more noticeable among the poorest quintiles [45, 46]. It indicates that household assets among poor families are more controlled by men who can access health facilities and health care. The poorest women may not, therefore, be able to use contraceptives of their choice and hence, be denied optimal family planning opportunities. Therefore, lower socioeconomic or wealth status of women is one key factor explaining low rates of modern contraceptive use. It also determines women’s low decision-making power to decide and access permission for contraceptive use and access health facilities [47, 48].

Overall, according to this study, poorer women were less likely to use transport and be controlled by their partners that resulted in not being able to access family planning services and use contraceptives. Additionally, this may be indirectly related to poor women having less access to education or media outlets to get a better understanding about the benefits of contraceptives. Gender wealth disparity, lack of access to any media, and limited permission to attend medical or health facilities are factors that negatively influence the use of contraceptives. These findings are consistent with other studies [49, 50]. It is clear that a higher prevalence of contraceptive use is found among women who have more knowledge, awareness, and media exposure. Our findings are consistent with these studies, as those women (aged 15–49) who have greater media exposure have more knowledge about contraceptive measures [51–53].

This study has shown the high disparity amongst women experiencing contraceptive utilisation living in rural and urban areas. Women living in rural areas were less likely to be exposed to contraceptive use than women who lived in urban setting settings. This finding may be attributed to women residing in urban areas having greater accessibility to family planning, and a better awareness about the benefits of family planning to prevent unintended pregnancy [2]. Moreover, legal practices and social protection are relatively better in urban settings in comparison to women living in a rural setting [2]. The community acceptance for male dominance in rural settings is relatively higher in comparison to women in urban areas and women are more empowered in urban residences; subsequently contributing to the utilisation of contraceptive use. This finding is consistent with a study conducted in rural India that demonstrated a lower contraceptive use and a higher experience of unintended pregnancy [54, 55]. Similarly, a study conducted in Ethiopia showed women in rural settings experienced higher unintended pregnancies and lower contraceptive use[23, 56]. In summary, it is important to consider and to distinguish that widespread education is primarily targeted to the individual, when greater structural changes (such as increased access to family planning in rural areas) may be more effective in eradicating and reducing IPV.

One of the interesting findings in this study showed that women whose household was headed by a male, were 1.4 times less likely to use contraceptives and this is associated with women’s disempowerment. Women negotiating the use of family planning is likely to occur less in households headed by males. Hence, increased power of decision-making on major household purchases, seeking health care services and attending public meetings were also positively influenced by the head of the household [47, 57]. Therefore, addressing women’s empowerment could provide a multipronged boost in the utilisation of contraceptives.

### Strength and Limitations of the Study

This study strength included the use of the standard measurement tools from DHS measures and large data sets from six Eastern SSA countries. It also used a rigorous analysis to identify the effects of IPV on intended pregnancy. The limitation of this study shows no causality of effects due to the nature of the cross-sectional design. Additionally, this study is only limited to DHS questionnaires and lacks some factors related to refugees, internally displaced people, and people who have travelled from other parts of the country, where IPV may be under reported. Another limitation of DHS surveys is subject to reporting and recall biases, the period of the DHS is not the same for all the countries and this also cause pose some challenges on the pooling of the datasets. Furthermore, the disadvantage of using a standardized questionnaire is that there are limited opportunities to adapt the questionnaire to be locally relevant. Additions, deletions and changes are made in every DHS survey, but the number of modifications is limited in order to maintain comparability, limit complexity of the survey, and keep the length of the questionnaire within limits.

## Conclusion

Women in Eastern SSA countries had a low rate of contraceptive utilisation and experienced higher rates of IPV. Physical violence was significantly positively associated with recent contraceptive use among married or cohabiting women in Eastern SSA. Additionally, recent contraceptive utilisation was associated with younger women, wealthier households, more educated women and exposure to various communication media, less barriers to accessing permission from the partner and less distances travelled to health care services. Hence, understanding how IPV affects women’s contraceptive use has important implications for ensuring that contraceptive access in the prevention programs can better meet the needs of women who experience IPV. Most importantly, minimizing the disparity with women and gender equality are important aspects to enhance contraceptive utilisation and prevention of IPV in Eastern SSA countries.

## Electronic supplementary material

Below is the link to the electronic supplementary material.

Supplementary Table A: Variables categorisation for identifying factor contraceptive non-use

## Data Availability

All the data available used are included in this study. Additionally, the original data from the survey are available online at measure DHS website.

## List of abbreviations

IPV: Intimate partner violence
DHS: Demographic and Health Surveys
aOR: Eastern SSA, adjusted odds ratios
OR: odds ratios
CI’s: confidence intervals
SDG: Sustainable Development Goals
ICF: Inner City Fund
Χ^2^: chi-square
UOR: unadjusted odds ratios
