# Supplementary material for: The association of intimate partner violence and contraceptive use: a multi-country analysis of demographic and health surveys
Source: Int J Equity Health. 2023 Apr 26;22:75. doi: 10.1186/s12939-023-01884-9 (PMC10134549; doi:10.1186/s12939-023-01884-9)
Supplement: Supplementary file 1 — Supplementary Table A: Variables categorisation for identifying factor contraceptive non-use [file 12939_2023_1884_MOESM1_ESM.docx]

| Supplementary Table A: Variables categorisation for identifying factor contraceptive non use | |
| --- | --- |
| **Variables** | **Variables name in the dataset categorisation** |
| contraceptive use in three categorical options “yes using any modern contraceptive method”; “yes using the traditional contraceptive method” and “never” | Contraceptive non-use (categorized as “used modern contraceptive method” and “never/ traditional contraceptive use”. Never or traditional contraceptive use was coded '1' and use of modern contraceptive was coded '0'). |
| IPV factors | |
| Any type IPV | It takes a binary form, such that Gender Based violence be regarded as a success (1 = IPV occurs in the specified age period) or failure (0 = if the IPV is not experienced). |
| Physical violence | Experience of physical violence coded as 0 " No" 1 "Yes" using the seven dimension of physical violence mentioned in the method section and having at least one of the seven question |
| Sexual violence | Experience of sexual violence coded as 0 " No" 1 "Yes" using the three dimension of sexual violence mentioned in the method section and having at least one of the seven question |
| Emotional violence | Experience of emotional violence coded as 0 " No" 1 "Yes" using the three dimension of emotional violence mentioned in the method section and having at least one of the seven question |
| Woman demographic factors | |
| Woman’s age | Mother’s age (1 = 15-24; 2 = 25-34; 3 = 35–49) |
| Woman’s age at first marriage | Woman’s age at first marriage (1 = 18 old and above ; 2 = less 18 years) |
| Age at first sexual experience | Mother’s age (1 = <15; 2 = 15-18; 3 = 18) |
| Woman’s education | Woman’s education ( 1=Secondary and above; 2= Primary; 3= No education |
| Woman’s working status | Maternal working status (1 = working; 2 = not working) |
| Current marital status | Currently Marital status 1 " Currently married" 2 "Formerly married" |
| Number of times married | Number of times married ( 1=one; 2 more than one) |
| Region denomination | Region denomination (1=Muslim ; 2=Catholic ; 3=Orthodox ; 3=Protestant; 4=Protestant; 5=Angelica; 6= Seventh; 7=pentecostal |
| Number of children ever born | Number of children ever born (1= two or less; 2=three to five; 3= greater than six |
| Knowledge of any type of family planning (FP) method | Knowledge of any type of family planning (FP) method ( 1= modern Methods; 2= Traditional or no methodss |
| Ever used FP Method | Ever used FP Method ( 1= Yes used at least one methods; 2= Never used) |
| Number of living children | Number of living children 1= two or less; 2=three to five; 3= greater than six |
| Household factors/ community and barriers to health care factors | |
| Head of household | Head of household (1=male; 2= female) |
| Wealth Index | Wealth Index (1= Rich ( very rich and rich); 2=middle; 3=Poor( very poor and poor) |
| Use of any of communications means | Use of any of communications means ( 1= if there at leat any of the media such as radio, TV or magazine reported; 2= No ) |
| Decision making power index | Categorised based the count measure using the three dimension below if a woman has reported 1= Full decision making power ( alone or jointly for all dimensions; 2=Considerable decision-making power ( if she has at least one dimension of decision making); 3= No decision-making power ( she has no power for any of the decision) |
| Women decision making- Purchase | Making decision on large household purchase( 1= woman alone; 2= Jointly, and 3= others including husband alone, some else or others) |
| Women decision making- family visit | Making decision on family visit ( 1= woman alone; 2= Jointly, and 3= others including husband alone, some else or others) |
| Women decision making- health care | Making decision to go to health care ( 1= woman alone; 2= Jointly, and 3= others including husband alone, some else or others) |
| Women reported being justified for violence | Women reported being justified for violence (1= if the women was not justified/accept any of the five dimension of beating; 2= if she accepts/justify one of the beating) |
| Attitude to wife beating: Justified if woman argues | Justified if woman argues with him 0 " No" 1 "Yes" |
| Attitude to wife beating: burns food, | burns food 0 " No" 1 "Yes" |
| Attitude to wife beating: burns food, | burns food 0 " No" 1 "Yes" |
| Attitude to wife beating: if she go out, | go out with telling her husband 0 " No" 1 "Yes" |
| Attitude to wife beating: if refuse to have sex | if refuse to have sex 0 " No" 1 "Yes" |
| Attitude to wife beating: if she neglect children care | if she neglect children care 0 " No" 1 "Yes" |
| Seek permission to visit health services | Seek permission to visit health services ( 1= no a big problem; 2= big problem) |
| Getting money to pay health services | Seek permission to visit health services ( 1= no a big problem; 2= big problem) |
| Travelling long distance to get health service | Travelling long distance to get health service ( 1= no a big problem; 2= big problem) |
| Hesitancy of attending health care alone | Hesitancy of attending health care alone ( 1= no a big problem; 2= big problem) |
| Place of residence | Place of residence ( 1= urban and 2= Rural) |
| Country | Country (1= Burundi,2= Ethiopia, 3=Kenya,, 4= Rwanda, 5= Uganda and 6= Tanzania) |
| Partners factors | |
| Partner’s age | Husband/ Partner age (1 =15-24; 2= 25-35; 3 =36-50; 4 =>50) |
| Partner’s working status | Current husband working status( 1=Working; 2 =not working) |
| Partner’s Education | Partner education ( 1=Secondary and above; 2= Primary; 3= No education) |
| Partner drinks alcohol | Partner drinks alcohol (0 = No; 1=Yes) |
| Father beat her mother | History of family beating (0 = No; 1=Yes) |
| Controlling Behaviour of partner | Controlling behaviour measured by six below questions how the partner controls his wife ( 0= if partners didn’t practice one of the below six measure; 2= if the partners practice at least one of the below question |
| Husband accuses her of unfaithfulness | Husband accuses for cheating ( 0 = No; 1=Yes) |
| Husband limit his wife contacting family | limit his wife contacting family ( 0 = No; 1=Yes) |
| Husband jealous | Husband jealous (0 = No; 1=Yes) |
| Husband insists on knowing where she is | Husband insists on knowing where she is (0 = No; 1=Yes) |
| Husband jealous | Husband jealous (0 = No; 1=Yes) |
| Husband not permit his wife to meet with friends | Husband not permit his wife to meet with friends (0 = No; 1=Yes) |
